# Pediatric medial epicondyle fractures with intra-articular elbow incarceration

**DOI:** 10.1007/s10195-014-0310-2

**Published:** 2014-07-26

**Authors:** Luigi Tarallo, Raffaele Mugnai, Francesco Fiacchi, Roberto Adani, Francesco Zambianchi, Fabio Catani

**Affiliations:** 1 Department of Orthopaedic Surgery, University of Modena and Reggio Emilia, Modena, Italy; 2 Department of Hand Surgery, University Hospital of Verona, Verona, Italy

**Keywords:** Medial epicondyle, Pediatric, Fractures, Incarceration, Outcome, Complications

## Abstract

**Background:**

Intra-articular incarceration of the epicondylar fragment occurs in 5–18 % of all cases of medial epicondyle fracture. It requires stable fixation to allow early motion, since elbow stiffness is the most common complication following medial epicondyle fracture. In this retrospective study, we report the clinical and functional outcomes and the complications that occurred following open reduction and screw fixation of medial epicondyle fractures with intra-articular fragment incarceration.

**Methods:**

Thirteen children who had a fracture of the medial epicondyle with incarceration of the fragment in the elbow joint (type III) were surgically treated in our university hospital between 1998 and 2012. There were eight male and five female patients. The mean age at the time of injury was 13 years (range 9–16). Operative treatment consisted of open reduction and internal fixation with one or two 4.0-mm cannulated screws under fluoroscopic control.

**Results:**

All of the patients were clinically reviewed at an average follow-up of 29 months. The overall range of motion limitation was about 5° for flexion–extension and 2° for pronation–supination. The score was excellent in all patients (mean 96.3). Complications occurred in four (31 %) children: two cases of symptomatic screw head prominence, irritation with partial lesion of the distal triceps myotendinous junction in one patient, and median nerve entrapment syndrome in one patient.

**Conclusions:**

In conclusion, open reduction and screw fixation yielded excellent clinical and functional outcomes for the treatment of medial epicondyle fractures with intra-articular fragment incarceration. However, particular attention is should be paid when treating these potentially serious injuries in order to minimize the risk of possible complications.

**Level of evidence:**

Therapeutic IV.

## Introduction

In the pediatric population, medial humeral epicondylar fractures account for nearly 12 % of all elbow fractures [1]. The medial epicondyle is the anatomic origin of the flexor carpi radialis, flexor carpi ulnaris, flexor digitorum superficialis, palmaris longus, part of the pronator teres, and the ulnar collateral ligament [2]. The major stabilizing ligamentous structure in the elbow is the anterior band of the ulnar collateral ligament; the posterior band only provides stability in flexion [3]. The fractured fragment is usually displaced distally due to traction forces exerted by its soft-tissue attachments [4].

There are three possible mechanisms of injury: a direct force applied to the medial epicondyle, an avulsive force from valgus or extension loading, and an association with elbow dislocation [5, 6].

Medial epicondyle fractures have been classified into four types depending on the extent of medial epicondyle displacement and the presence of a concomitant: a small degree of avulsion (type I); a non-entrapped avulsed fragment at the level of the joint (type II); a fragment incarcerated in the joint (type III); a fracture associated with elbow dislocation (type IV) [7].

Whereas previous studies have recommended open reduction and internal fixation when the epicondyle is displaced by >2–5 mm [8, 9], numerous studies have recently reported that nonsurgical treatment yields results that are similar to or better than those of surgical treatment [10, 11].

Current absolute indications for open reduction and internal fixation of medial epicondylar fractures include incarceration of the epicondylar fragment in the elbow joint, suspected entrapment and dysfunction of the ulnar nerve, marked instability, and open fracture [12]. Moreover, the surgical treatment must be taken into account in cases of high-energy trauma, elbow laxity or instability, and significant fracture displacement [11].

Intra-articular incarceration of the epicondylar fragment occurs in 5–18 % of cases [13] and requires stable fixation to allow early motion, since elbow stiffness is the most common complication following medial epicondyle fracture [14]. In the study reported in the present paper, we evaluated the clinical and functional outcomes and the complications that occurred following open reduction and fixation with screws of medial epicondyle fractures with intra-articular fragment incarceration.

## Materials and methods

Thirteen children who had a fracture of the medial epicondyle with incarceration of the fragment in the elbow joint (type III) were surgically treated in our University Hospital between 1998 and 2012. All the fractures were closed and resulted from a fall on the outstretched hand. Four cases were associated with a posterolateral elbow dislocation.

There were eight male and five female patients. The dominant arm was involved in eight children. The age at the time of injury ranged from 9 to 16 years, with an average of 13 years.

Standard anteroposterior and lateral plain films of the injured elbow were obtained preoperatively for all patients.

**Fig. 1:**
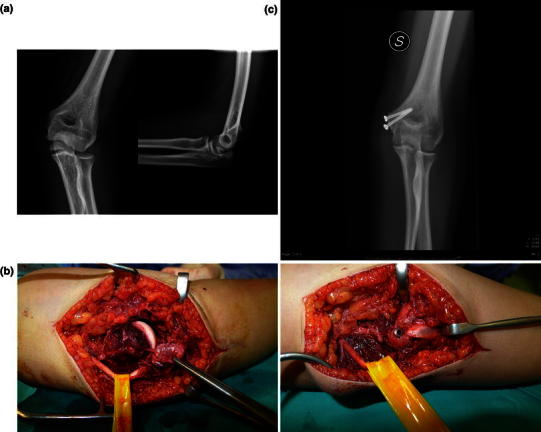
Case 1.**a** X-ray showing medial epicondyle fracture with intra-articular fragment incarceration.**b** Intraoperative view of ulnar nerve identification and protection followed by open reduction and internal fixation with two cannulated screws.**c** X-ray at 3 months, showing complete healing of the fracture

The operations were performed under general anesthesia with the patient in the supine position and the injured elbow on an arm board. Operative treatment consisted of open reduction and internal fixation with a 4.0-mm cannulated screw under fluoroscopic control. When the epicondylar fragment was large enough, a second screw was used to provide rotational stability. The screws were placed up the medial column of the elbow, avoiding the olecranon fossa. The medial epicondyle was exposed using a medial longitudinal incision. The ulnar nerve was routinely identified and protected but not transposed (case 1, see Fig. 1a–c). Postoperatively, patients were immobilized with a cast at 90° flexion and with the forearm in neutral rotation for 2 weeks. Patients were then placed in a posterior splint and encouraged to remove the splint to perform gentle passive and active range-of-motion exercises 3–5 times per day. The splint was removed after pain-free palpation of the medial epicondyle, usually at 1 month after surgery.

It is our routine practice to clinically evaluate all patients at 2 weeks and perform both clinical and radiological evaluations at 1 and 3 months. Moreover, we organized an additional clinical follow-up in September 2013.

The postoperative clinical evaluation was performed by one of the authors and included analysis of passive and active range of motion (ROM), functional results based on the Mayo Elbow Performance Score (MEPS) [15], pain levels during activities of daily life evaluated with a 10 cm Visual Analogue Scale (VAS) [16], elbow stability, and early or late complications. Flexion–extension of the elbows and pronation–supination of the forearm were measured by a goniometer. The uninjured elbows served as controls.

We decided to use the MEPS as it can be completed quickly, it assesses elbow function and pain via questions and elbow condition via objectively measured clinical data, and all of its items are applicable to pediatric subjects. The total MEPS score ranges from 5 to 100 points, with higher scores indicating better function. If the total score is between 90 and 100 points, it can be considered excellent; between 75 and 89 points, good; between 60 and 74 points, fair; less than 60 points, poor [15]. The stability of the elbow was evaluated with a manual valgus stress test at 15° of flexion.

Possible early or late complications were assessed and recorded at each follow-up evaluation.

## Results

All of the patients were clinically reviewed an average follow-up of 29 months. X-rays showed solid union in all patients. At the final examination, all of the children presented an excellent range of motion. The overall ROM limitation was about 5° for flexion–extension and 2° for pronation–supination. The MEPS score was excellent in all children (mean 96.3, range 90–100).

**Fig. 2:**
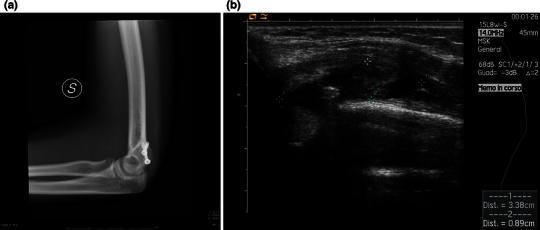
Case 2.**a** X-ray showing a screw tip slightly protruding posteriorly from the bone surface.**b** Ultrasound examination showing the presence of a hematoma with a partial lesion of the myotendinous junction of the triceps over the protruding screw tip

**Fig. 3:**
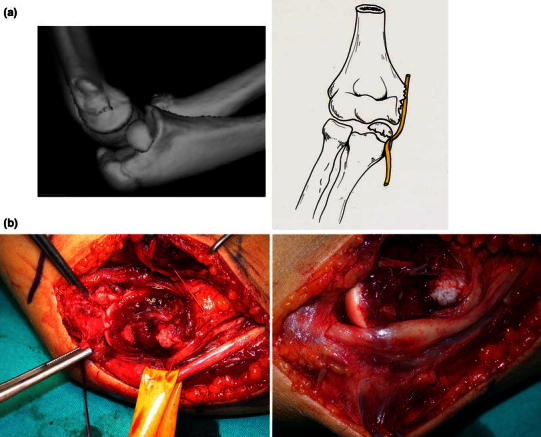
Case 3.**a** Preoperative CT study with reconstruction showing the median nerve entrapped within the joint by the fragment and the medial collateral ligament after the trauma.**b** Intraoperative view showing median nerve release after osteotomy of the previously fractured fragment, with the presence of swelling at the site of compression

Complications occurred in four (31 %) patients. There were three cases of screw removal due in two cases to symptomatic screw head prominence and in one case to irritation with partial lesion of the distal triceps myotendinous junction caused by the protrusion of the screw tip posteriorly, causing impingement of the triceps tendon during elbow flexion–extension. The latter case was completely asymptomatic for the first 4 months after surgery, but the patient complained of pain during elbow flexion–extension after resuming sporting activity (swimming). The clinical examination revealed the presence of a painful swelling at the distal third of the humerus. The lateral X-ray projection showed that one screw was oriented posteriorly with the screw tip protruding slightly from the bone surface, and echography demonstrated a partial lesion of the myotendinous junction over the protruding screw tip (case 2, see Fig. 2a, b). After screw removal and splint immobilization for 2 weeks, complete recovery and pain relief were reported. Moreover, we observed persistent median nerve symptoms (anterior interosseous nerve syndrome with weakness of the flexor pollicis longus and flexor digitorum profundus muscles associated with pain centered over the antecubital fossa and extending distally into the proximal forearm) after surgery in one case associated with posterolateral elbow dislocation. In this case, the median nerve was entrapped within the joint by the fragment and the medial collateral ligament after the trauma. The median nerve was not explored during surgery and remained entrapped within the joint (case 3, see Fig. 3a). The patient underwent a second surgery consisting of osteotomy of the previously fractured fragment, median nerve release (case 3, see Fig. 3b), and new fixation with one cannulated screw, leading to relief from symptoms within 2 months. No other neurological complications were observed. Pain during activities of daily life was absent in all patients at the final clinical evaluation, except in the patient who was re-operated on for median nerve entrapment.

No patient had elbow instability or valgus deformity. All patients resumed their sporting activities at a mean 4 months after surgery, and all patients returned to their previous level of activity (Table 1).

**Table 1:** Patient details, clinical outcome at the latest follow-up, and complications

Range of motion (°)										
Patient	Age	F.U. (months)	Δ Flexion	Δ Extension	Δ Pronation	Δ Supination	Pain during activities (VAS)	MEPS	Time until sporting activities were resumed (months)	Complications
1	10	27	0	0	0	0	0	100	3	
2	13	33	3	5	0	0	0	100	3	
3^a^	11	28	0	2	0	2	0	100	4	
4^a^	13	37	5	0	0	0	2	92	8	Median nerve intra-articular entrapment
5	9	25	0	0	3	3	0	92	2	
6	12	18	3	10	2	5	0	90	6	Pain (screw removal)
7	14	27	2	0	0	0	0	98	3	
8	16	29	7	5	2	2	0	93	2	
9^a^	15	35	0	3	0	5	0	94	6	
10	13	33	5	0	0	0	0	100	2	
11	15	31	0	0	0	0	0	98	2	Partial lesion of the distal triceps myotendinous junction (screw removal)
12^a^	10	22	2	7	0	0	0	95	3	
13	14	26	0	0	0	0	0	100	4	Pain (screw removal)

^a^ Cases associated with posterolateral elbow dislocation

## Discussion

Many authors agree that fractures of the medial epicondyle with incarceration of the fragment in the elbow joint (type III) should be surgically treated [12, 17–20]. Multiple methods of surgical treatment have been reported: fragment excision and sutures [10, 21], closed reduction and percutaneous Kirshner wires [22], open reduction and Kirshner wires [10, 23–25], open reduction and sutures [6, 9, 26], open reduction and smooth pins [9, 27], and open reduction and screws [11, 25, 28]. The goals of operative fixation are to maximize the possibility of early return to full function and high-level activity and to minimize late deformity and the likelihood of stiffness (as with prolonged cast immobilization). Therefore, the fracture fixation method employed must be secure enough to allow for early elbow mobilization [29]. Lee et al. stated that operative treatment with suture fixation is unstable and requires supplementary immobilization with a splint; K-wire fixation provides improved stability over sutures, but supplementary splint immobilization is also required [25]. Furthermore, if motion is attempted with Kirshner wire fixation, the wires tend to bind the skin and inhibit early ROM [29].

Moreover, Kamath et al. suggested in their systematic review that the use of Kirshner wires or smooth pins for fixation could not achieve adequate compression, leading in some cases to bony nonunion [30]. However, a potential drawback of screw fixation is the symptomatic prominence of the screw head over the epicondyle, which produces irritation that sometimes requires the removal of the hardware [9, 31]. Another factor that should be taken into account in the choice of the surgical technique is the patient’s age. In fact, it has been suggested that the ratio of elbow growth to width has the same biomechanical importance as longitudinal growth in terms of muscle balance and stability [32]. Therefore, in very young patients, K-wire fixation should be preferred, since screws should be routinely removed to avoid growth anomalies [28, 33].

In the present research, open reduction and internal fixation with one or two cannulated screws provided stable fixation, leading to a 100 % rate of bony union and resulting in excellent functional and clinical outcomes in all patients, with early resumption of sporting activities. Our functional results are in line with those reported by Lee et al., who obtained good to excellent results at a mean follow-up of 27.2 months when evaluations were performed based on the Elbow Assessment Score of the Japanese Orthopedical Association in all surgically treated patients. In particular, the mean score was 97.1 points in patients who received screw fixation, 96.3 for those who received Kirshner wire fixation, 94.5 points after tension-band wire fixation, and 93.5 following interosseous suture [25]. When the ROM evaluations were considered, we calculated a mean loss of about 5° for flexion–extension and 2° for pronation–supination. Several studies in the literature evaluated the ROM in patients who had been surgically treated for medial epicondyle fractures. However, different methods of fixation were evaluated at the same time in these studies, and different fracture types were often included. In particular, Pimpalnerkar et al. found a mean loss of extension of 6.4° (range 0–15) and a mean loss of supination of 2.5° (range 0°–10°) in patients with type IV fractures treated with either Kirshner wires or screws [23]. Duun et al. reported that seven of their 33 surgically treated patients lost extension (5°–25°), one lost supination (10°), and two lost flexion (5°) [9]. Louahem et al. retrospectively evaluated 139 patients who were surgically treated with Kirshner wires in 129 cases and compressive screws in 10 cases, and reported normal elbow ROM at a mean follow-up of 3.9 years in 133 patients. The six remaining (three with a type III and three with a type IV fracture) had extension deficits of <20°. The final clinical result was excellent in 130 patients and good in nine [24].

Complications, including hardware removal, were documented in four (31 %) children. Painful screw head prominence was reported in two subjects, and irritation with partial lesion of the distal triceps myotendinous junction caused by the protrusion of the screw tip posteriorly was reported in one subject. This case suggests that particular attention must be paid when inserting the screw, as it must be placed up the medial column of the elbow, avoiding the olecranon fossa, and any eventual screw tip protrusion must be checked for by monitoring different fluoroscopic projections.

Moreover, we reported a case in which the median nerve was not explored during surgery; it remained entrapped within the joint, with consequent median nerve entrapment syndrome observed. Therefore, it is important to perform neurolysis of the nerve in addition to surgical exploration, particularly in the most complex fractures—especially those associated with elbow dislocation.

In conclusion, open reduction and screw fixation proved excellent clinical and functional outcomes for the treatment of medial epicondyle fractures with intra-articular fragment incarceration. However, particular attention must be paid when treating these potentially serious injuries in order to minimize the risk of possible complications.
